# Neuro-Adipokine Crosstalk in Alzheimer’s Disease

**DOI:** 10.3390/ijms25115932

**Published:** 2024-05-29

**Authors:** Bandy Chen, Marc Schneeberger

**Affiliations:** 1 Laboratory of Neurovascular Control of Homeostasis, Department of Cellular and Molecular Physiology, Yale School of Medicine, New Haven, CT 06510, USA; bandy.chen@yale.edu; 2 Wu Tsai Institute for Mind and Brain, Yale University, New Haven, CT 06510, USA

**Keywords:** adipokines, aging, Alzheimer’s disease, neurodegeneration, obesity

## Abstract

The connection between body weight alterations and Alzheimer’s disease highlights the intricate relationship between the brain and adipose tissue in the context of neurological disorders. During midlife, weight gain increases the risk of cognitive decline and dementia, whereas in late life, weight gain becomes a protective factor. Despite their substantial impact on metabolism, the role of adipokines in the transition from healthy aging to neurological disorders remains largely unexplored. We aim to investigate how the adipose tissue milieu and the secreted adipokines are involved in the transition between biological and pathological aging, highlighting the bidirectional relationship between the brain and systemic metabolism. Understanding the function of these adipokines will allow us to identify biomarkers for early detection of Alzheimer’s disease and uncover novel therapeutic options.

## 1. Fat as a Clinical Feature in Neurological Disorders

Neurological disorders are characterized by overall impairments in energy homeostasis and uniquely by their specific disease markers associated with the central nervous system (CNS). With the rapidly aging population, an urgent need is to uncover novel approaches for preventing and treating neurological disorders. This requires the complementation of peripheral markers to canonical central markers of disease. Obesity is a risk factor for various neurological disorders, with an altered adipose tissue milieu as a common denominator. Recent studies highlight the role of peripheral organs, particularly the white adipose tissue (WAT), in shaping brain structure and function [1,2]. Understanding the role of adipose tissue in the development and progression of CNS disorders provides novel therapeutic approaches to treat the ever-increasing neurodegenerative population.

Alzheimer’s disease (AD) is the primary cause of dementia, with age being the main risk factor [3]. The incidence rate doubles approximately every 5 years among individuals aged 65 years and older [4]. AD pathogenesis involves the accumulation of amyloid beta (Aβ) and tau protein, leading to the formation of amyloid plaques and neurofibrillary tangles, respectively [5]. While cognition and memory deficits are the primary clinical features of AD, body weight alterations are secondary features linked to disease progression. Midlife weight gain increases the risk of AD, with obesity-related brain changes resembling those seen in AD [6,7]. Conversely, late-life weight gain may protect against the onset and progression of AD [8,9]. However, most studies utilize body mass index (BMI) as a predictive measure, which does not accurately reflect regional adiposity [10]. Understanding the role of specific regional fat depots and their depot-specific secretome will allow better characterization of the neuro-adipose crosstalk in biological and pathological aging [11]. The conundrum in weight fluctuations underscores the importance of investigating the structure and function of WAT in relation to the prevention and treatment of AD.

## 2. Remodeling of Adipose Tissue Shapes Cognitive Function

The association between midlife weight gain and cognitive decline have shed light on the brain–body crosstalk in the regulation of brain health. Chronic overnutrition dramatically changes the metabolic profile of the body, altering levels of nutrients, metabolites, and hormones. In an obesogenic environment, the adipose tissue expands to compensate for the excess nutrients, recruiting immune cells and transitioning to an inflammatory milieu [12]. Excessive adiposity is a risk factor for cognitive decline due to a rewiring of whole-body metabolism [13]. The mechanisms of the brain–adipose axis in modulating cognitive function remains to be elucidated. A study screening human adipose tissue identified genes that are linked to cognitive performance [14]. Manipulation of these genes demonstrated improvements in cognitive performance for various models including rodents and flies. In line with these results, visceral adipose tissue (VAT) NLPR3 inflammasome impairs cognitive function through an IL-1-mediated microglial activation [15]. The proposed mechanism involves VAT NLRP3 induction of IL-1β secretion from local macrophages, with elevated peripheral IL-1β triggering an inflammatory response in brain-resident immune cells. Whether peripheral IL-1β further recruits peripheral immune cells to the brain remains to be elucidated. Adipose tissue-derived mesenchymal stem cells (ADSCs) have been shown to improve both cognitive function and physical activity in aging mice [16]. However, in the context of obesity, ADSCs can be distinguished into two categories: pro-inflammatory and anti-inflammatory. The pro-inflammatory ADSCs can increase neuroinflammation by inducing proliferation and differentiation of CD4+ and CD8+ T cells [17]. Transplantation of ADSCs into a rat model of AD ameliorates cognitive impairments, enhances Aβ clearance, and suppresses apoptosis and neuroinflammation by modulating central and systemic SIRT1 levels [18,19]. Intraventricular injection of ADSCs has been shown to improve AD pathogenesis by modulating central cholesterol homeostasis [20]. A detailed review focusing on the role of ADSC-related therapy in CNS disorders is found elsewhere [21]. Adipose tissue-derived extracellular vesicles and microRNAs in obese patients and rodents cause synaptic loss and cognitive impairment [22]. These molecules enter the brain via a membrane protein-dependent manner and demonstrate a preference for neurons. In the obese brain, these molecules are enriched in the hippocampus and hypothalamus, likely contributing to the rewiring of higher-order processing and satiety regulation, respectively [22]. Diabetes mellitus induces abnormal expression of exosome miRNA in multiple peripheral tissues with an altered central exosome absorption [23]. One possibility is that there is an increase in exosome miRNA flux into the brain that leads to a loss of aquaporin-4 expression and redistribution in perivascular astrocyte endfeet, resulting in glymphatic dysfunction and cognitive decline [23]. Downregulation of these extracellular vesicles ameliorates cognitive impairments, thereby making them novel targets for pharmaceutical intervention to treat metabolically induced cognitive dysfunction. These studies support the role of a brain–adipose axis in the regulation of cognitive function, with alterations in the adipose tissue milieu as a driver of cognitive decline. Further investigation into an altered adipose tissue milieu in chronic overnutrition can help explain the transition from biological to pathological aging and provide a link between obesity and neurological disorders.

## 3. Change in Adiposity across Biological and Pathological Aging

The increasing aging population has led to an unprecedented level of chronic metabolic and neurodegenerative diseases. Throughout aging, the body transitions from an anabolic state to a catabolic state, resulting in overall weight loss. Before such weight loss is manifested, most individuals experience an increase in adipose tissue mass due to overnutrition, reduced physical activity, and a lowered basal metabolic rate [24,25]. This weight gain throughout early to middle adulthood increases the risk of major chronic diseases and mortality [26]. Late midlife adiposity is a proposed predictor of frailty in late life [27]. On the other hand, age-related weight loss can stem from various factors, including undernutrition, sarcopenia, and cachexia [28]. Importantly, weight loss in late life is linked to a heightened risk of mortality, irrespective of baseline weight [24]. Further research on the composition of the adipose tissue milieu throughout the transition from midlife weight gain to late-life weight loss will provide insight on its role in biological and pathological aging. Whether the initial transition of weight change stems from a central or peripheral root cause remains to be elucidated. A fundamental question that remains unanswered is whether weight loss itself exacerbates mortality or whether it is a consequence of pathological diseases that subsequently increase mortality.

**Figure 1:**
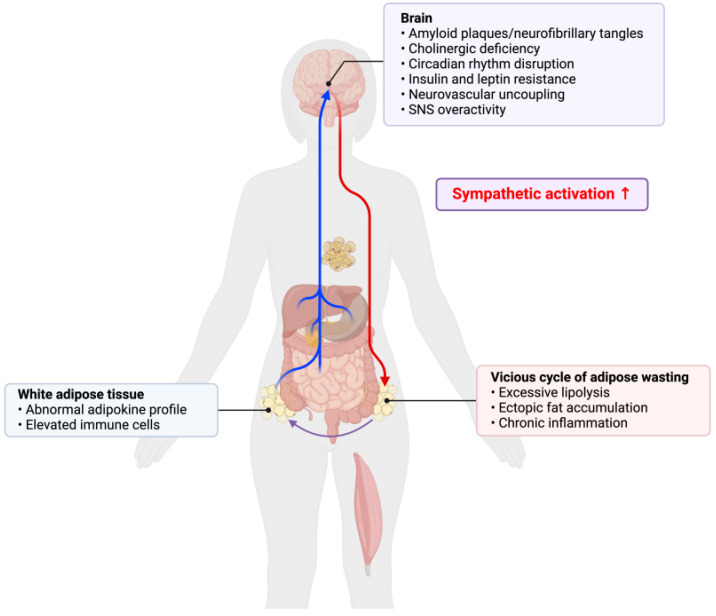
SNS overactivity in Alzheimer’s disease. Impaired metabolic profile and central circuit rewiring results in an elevated sympathetic tone. This leads to a catabolic state, driving adipose tissue lipolysis with elevated free fatty acids further promoting positive feedback. Figure created using Biorender.

While weight loss is a natural outcome of biological aging, excessive weight loss serves as a clinical manifestation of AD [29]. The weight loss seen in AD is attributed to multiple factors, including lower energy intake and higher resting energy expenditure [30]. Patients diagnosed with mild cognitive impairment (MCI) who experience weight loss have an elevated risk of developing AD [31]. Similarly, individuals with AD who undergo weight loss are at a heightened risk of disease progression, leading to increased morbidity and mortality [32]. There is a positive association between cognitive performance and sarcopenic obesity in older adults with AD [33]. The occurrence of late-life weight loss precedes the onset of cognitive decline in AD [34,35]. An association between late-life weight loss and the development of MCI is found independent of midlife body weight [36]. Interestingly, recent data suggest that both substantial late-life weight gain and weight loss are associated with a higher risk of dementia [37]. Hence, it is plausible that significant shifts in either direction of the body weight set point leads to a decline in brain health due to improper compensatory mechanisms. This raises the questions of whether an altered adipose tissue milieu precedes cognitive decline in both biological and pathological aging, and what are the peripheral messengers involved in the brain–body crosstalk to mediate this shift. One possible explanation that connects the systemic alterations seen in AD is dysautonomia, a state of autonomic nervous system dysfunction [38]. AD is characterized by cholinergic dysfunction, resulting in a chronic state of neuronal hyperactivity [39]. Levels of cholinergic receptor binding are inversely associated with the severity of dementia [40]. Excess sympathetic tone results in an overall catabolic state. For example, increased sympathetic innervation to the WAT leads to elevated lipolysis, with elevated free fatty acids resulting in ectopic accumulation of lipids and contributing to a chronic inflammatory state (Figure 1). This explains the generalizing wasting syndrome associated with AD [41]. The miscommunication between the brain and adipose tissue, resulting in autonomic dysfunction, is also reported in cancer-associated cachexia [42]. Further investigation into the somatosensory innervation of adipose tissue can assist in elucidation of the neuro-adipose crosstalk in health and disease [43] Though late-life BMI is inversely correlated with AD risk, future studies comparing late-life weight maintenance and weight gain are needed to assess the therapeutic efficacy of late-life overnutrition. Whether midlife weight gain or late-life weight loss is a greater risk factor to higher morbidity and mortality of neurological disorders needs clarification.

## 4. Adipokines as Mediators between the Brain and Body

As an endocrine organ, WAT consists of preadipocytes, mature adipocytes, mesenchymal cells, and various other cell types that constitute the stromal vascular fraction [44]. Hundreds of cytokines are secreted from WAT and have been identified and termed as “adipokines”. Adipokines have been implicated in several aspects of brain metabolism such as leptin in food intake regulation and adiponectin in brain glucose metabolism [1]. Structural and functional alterations of WAT occur throughout biological aging and influence the quantity and type of adipokines secreted [45]. Aging is marked by an escalation in VAT, driven by chronic positive energy balance and a shift in lipid deposition from the subcutaneous to the visceral fat depot [46]. The expansion of VAT is linked to an increase in proinflammatory adipokines and a decrease in anti-inflammatory mediators [45]. Both generalized and VAT are associated with reduced cognitive function, after adjustment for cardiovascular risk factors and vascular brain injury [13]. Interestingly, subcutaneous WAT and omental WAT are associated with different plasma markers and cerebrovascular health in severely obese patients [47]. While subcutaneous WAT demonstrates more crown-like structures, indicative of inflammation and hypertrophy, there is no association between subcutaneous WAT parameters and brain health. On the other hand, omental WAT is positively related to greater variability in CBF, indicating vascular and perfusion abnormalities, in the parietal lobe and nucleus accumbens [47]. Furthermore, aging is associated with the accumulation of ectopic fat, which is correlated with an increased risk of cardiometabolic disorders [48,49]. Increased ectopic fat accumulation is associated with cognitive impairments, decreased total brain volume, and increased lateral ventricle volume [50]. In rodents fed with a high-fat diet (HFD), visceral adiposity links cerebrovascular rewiring to cognitive impairments [51]. To sustain metabolic homeostasis, maintaining a balanced proportion of both total adiposity and regional distribution is crucial [52]. Central fat distribution and the relative loss of fat-free mass are more accurate than BMI in determining various health risks associated with biological and pathological aging [53]. Higher VAT metabolism is linked to greater brain Aβ burden in older subjects with cognitive impairment [54]. Future studies differentiating adipose tissue composition in AD will help explain the difference in adipokine profiles. Discerning both plasma and cerebrospinal fluid (CSF) levels of adipokines in biological aging compared to pathological aging will provide insights into the molecular mechanisms behind body weight alterations and systemic metabolism in AD. In this review, we explore the potential roles of key adipokines in the development and progression of AD. We aim to offer fresh insights on the involvement of adipokines in AD and unveil potential avenues for the discovery of novel therapeutic targets and diagnostic tools. Given the abundance of adipokines implicated in neurometabolism, we focus on those that are extensively researched.

## 5. Altered Adipokine Profile in Biological and Pathological Aging

**Figure 2:**
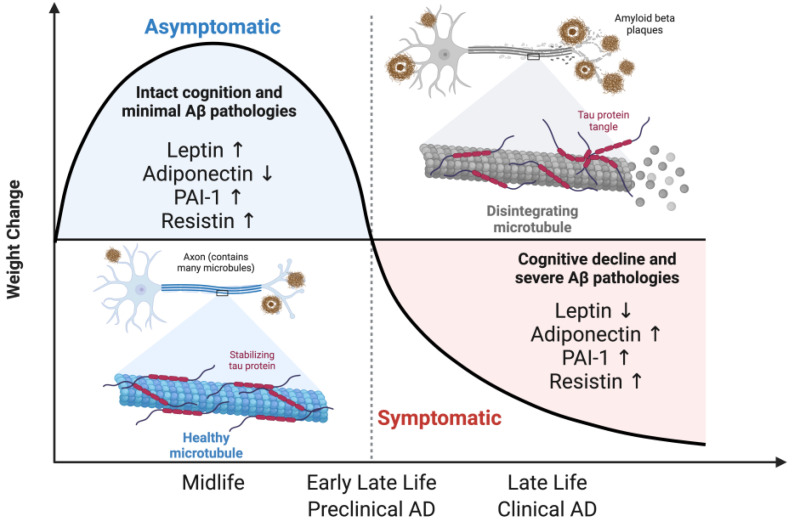
Alzheimer’s stages in the context of adipokines. The transition from midlife to late life leads to weight loss and alterations in the structure and function of adipose tissue. This leads to an altered adipose tissue secretome, resulting in a pathological adipokine profile that enhances AD pathology. Figure created using Biorender.

Leptin is secreted in proportion to the amount of adipose stores in the body [55]. Its primary role is to communicate to the brain on the status of peripheral energy storage levels, making it a critical adipokine for the maintenance of body weight and a hallmark of obesity. In addition to its metabolic roles, leptin is involved in the maintenance of a proper cerebral landscape. Both leptin and leptin-receptor-deficient mice demonstrate remodeling of the cerebrovascular architecture and gliovascular unit [56,57]. Peripheral nerve regeneration requires Schwann cell leptin receptor signaling, indicating a role for leptin in myelination [58]. Administration of leptin in the leptin-deficient ob/ob mouse model ameliorates hypomyelination [59]. Additional studies are required to differentiate whether these benefits are independent from body weight changes. Leptin ameliorates AD pathology by targeting multiple steps in the Aβ cycle, including production, clearance, degradation, and aggregation [60,61,62]. Decreased plasma leptin levels are correlated with an increased risk of cognitive decline and AD [63]. Plasma leptin levels are inversely correlated with CSF Aβ, with lower plasma leptin levels indicating greater brain Aβ deposition [64]. Conversely, higher levels of leptin in the elderly are shown to be protective against cognitive decline, independent of comorbidities and body fat [65]. The decline in leptin levels in AD patients is correlated with the severity of dementia symptoms and changes in body weight [64,66] (Figure 2). A study indicates that higher plasma leptin levels are associated with reduced risk of AD only in non-obese patients [65]. Additionally, higher leptin levels are associated with enhanced cognitive performance among normal-weight participants, while no such association was observed in overweight individuals [67]. A potential explanation is that the development of leptin resistance in obese patients during midlife diminishes the effectiveness of leptin in late life. Although weight loss is associated with increased leptin sensitivity, chronic overnutrition in midlife could lead to an irreversible state of leptin resistance. It remains unclear whether elevated leptin levels are actively involved in the development of obesity or are markers of metabolic dysfunction due to chronic overnutrition. Whether leptin levels and sensitivity change throughout biological aging independent of adiposity remains to be elucidated. Overall, these findings suggest that low leptin levels could serve as an early biomarker for identifying AD, and the potential to leverage leptin as a therapeutic agent to treat AD pathology.

Adiponectin is involved in insulin sensitization and modulation of anti-inflammatory and antioxidant activities [68]. It has been shown to mediate myelination by reducing myelin lipid accumulation in myelin-laden macrophages, mitigating neuroinflammation [69,70]. Plasma adiponectin levels increase with age in both men and women [71]. Unlike many adipokines, the expression of adiponectin inversely correlates with adiposity [72]. Reduced levels of adiponectin are correlated with metabolic syndrome [73]. In young adults with metabolic syndrome, adiponectin levels are positively correlated with total macrovascular CBF. Paradoxical to plasma leptin levels, midlife obesity results in lowered plasma adiponectin levels, while late-life weight loss results in elevated adiponectin levels [74] (Figure 2). Interestingly, greater adiponectin levels in early life are associated with cardioprotective benefits; however, in midlife and late life, elevated levels are associated with poor cardiovascular outcomes [71]. In late life, elevated adiponectin levels are associated with reduced physical functioning and greater all-cause mortality [75]. Elevated serum adiponectin levels and insulin resistance is reported in AD patients compared to MCI patients and healthy controls [76,77]. This positive correlation supports the existing theory of adiponectin resistance in AD. This is further supported by correlations between adiponectin levels and dementia [78]. In MCI subjects, CSF adiponectin levels are associated with cortical glucose metabolism [79]. Additionally, plasma adiponectin levels are linked to the rate of cognitive decline and cortical thinning in Aβ (+) MCI [80]. Impaired central adiponectin signaling likely contributes to brain insulin resistance and altered glucose metabolism seen in AD. Whether adiponectin directly targets known AD markers such as Aβ and tau is unclear. Considering the isoforms of adiponectin, serum levels of different molecular weights have diverse implications for cognitive decline and AD. Future studies concentrating on quantifying adiponectin isoforms in biological and pathological aging will result in greater predictive value of AD diagnosis. It is unclear whether adiponectin signaling is impaired in these studies, reinforcing a need to measure adiponectin receptors and downstream signaling molecules in conjunction with insulin and insulin receptors with a temporal component to better pinpoint the development of adiponectin resistance. A hypothesis to explain the paradoxical relationship between high adiponectin levels throughout aging is that elevated levels are likely a compensatory response to maintain redox homeostasis due to the systemic chronic inflammation occurring in biological aging [81]. This compensatory response is exacerbated in AD, where in addition to inflammatory aging markers, greater levels of adiponectin are secreted in response to AD pathologies. Overall, the utilization of plasma adiponectin levels as an AD predictor remains premature due to the heterogeneity of clinical studies, likely attributed to the different timepoints of biological and pathological aging [82].

Plasminogen activator inhibitor-1 (PAI-1) is a serine protease inhibitor primarily linked to thrombosis and fibrinolysis, with other biological roles including regulation of cell migration, tissue remodeling, and angiogenesis [83]. Plasma levels of PAI-1 exhibit a positive correlation with weight gain, and elevated levels are associated with metabolic syndrome and obesity [84]. Considering that obesity is a main driving force for the incidence of stroke, alterations to PAI-1 levels and signaling are proposed as a key nexus that explains the mechanistic link between obesity and stroke [85]. This link can be extended to explain the metabolically induced transition from biological aging to pathological aging. Aging correlates with increased levels of PAI-1, with aged tissues displaying greater levels of PAI-1 expression [86]. An increase in PAI-1 levels correlates with cognitive decline, and patients with AD exhibit elevated levels [87]. Plasma PAI-1 levels gradually increase with dementia progression, suggesting the potential of PAI-1 as an early indicator of AD [88]. Whether PAI-1 directly contributes to AD pathology or serves as a compensatory mechanism remains unanswered. As an inhibitor of plasminogen, elevated levels of PAI-1 are associated with impaired Aβ clearance [89,90]. One possibility is that the initial accumulation of Aβ preceding cognitive decline and dementia triggers an increase in plasmin activation and a decrease in PAI-1 levels to reduce Aβ levels. The decline in PAI-1 levels observed in early late life (preclinical AD) corresponds to early late-life weight loss, as PAI-1 strongly correlates with BMI and adiposity [91]. The subsequent increase in PAI-1 seen in late life (clinical AD) may be attributed to Aβ formation, further worsening AD pathology (Figure 2). As an upstream regulator of BDNF, the PAI-1/BDNF ratio is proposed as a selective marker of AD patients with full dementia to distinguish from other prodromal AD stages and healthy controls [87]. This reinforces the concept of using multiple markers and the ratios between them as indicators of disease progression, instead of solely relying on a single marker or disconnected individual markers. Longitudinal studies that focus on the changes in PAI-1 levels during the preclinical and clinical stages of AD are necessary to elucidate the role of PAI-1 in AD.

Resistin is a cysteine-rich secretory protein that counteracts insulin action and impairs glucose homeostasis [92]. Plasma resistin levels are positively correlated with weight gain, and link obesity to diabetes [93]. Elevated levels of resistin increase the risk of developing cardiovascular disease and insulin resistance [94]. Acute cerebral infarction patients exhibit higher serum resistin levels, which indicate that serum resistin levels may be a risk factor for pathological cerebrovascular remodeling [95]. In elderly patients, higher levels of resistin are associated with greater risk for all-cause mortality, independently of cardiovascular risk factors [96]. Both plasma and CSF levels of resistin are elevated in AD patients compared to healthy controls [97,98] (Figure 2). This correlation extends to other cognitive impairments such as MCI and all-cause dementia [99,100,101]. Interestingly, resistin attenuates oxidative stress induced by Aβ [102]. Conversely, treatment with resistin exacerbates Aβ pathology in a rodent model of AD and metabolic syndrome [103]. In the same rodent model, treatment with adiponectin ameliorates glucose metabolism and cognitive function and decreases Aβ load. A direction to explore is the interaction between adiponectin and resistin on brain insulin signaling and glucose metabolism, as both seemingly have opposing functions on AD pathology [103]. One hypothesis is that acute elevated levels of resistin is a compensatory response to the accumulation of Aβ; however, chronic levels lead to both brain and peripheral insulin resistance and worsen AD pathology. This explains why simultaneous exposure to both HFD and resistin greatly worsens Aβ pathology compared to HFD alone [103]. Studies tracking resistin levels throughout the different stages of AD is required to determine the beneficial and detrimental levels of resistin.

Circulating levels of adipokines are promising candidates that can complement known markers of AD pathology to enhance the monitoring of AD. It is imperative for future studies to measure a variety of adipokines rather than biasedly picking a few to establish correlation with cognitive performance and AD pathology. These adipokines act dependently on each other and with known AD markers; therefore, utilization of multiple markers is required to accurately determine the stages of AD. The distinction between early late life (preclinical AD) and late life (clinical AD) requires more nuance due to the different phenotypes and interindividual variability, which can explain the opposing results in biomarker measurements seen in AD studies.

## 6. Adipokines as Therapeutic Agents for Neurometabolic Dysfunction

With the exponential rise in neurological disorders, it puts pressure on the healthcare field to come up with novel therapeutic strategies. The potential of using adipokines as therapeutic agents has been explored in the context of peripheral disorders often associated with obesity [104]. A mechanism through which mesenchymal stem cell therapy improves metabolic health is the modulation of adipokine profiles [105]. With the growing link between obesity and neurological disorders, the field of neurodegenerative treatment will benefit from viewing neurological disorders with a metabolic lens [106]. Evidently, changes in adipokine profiles are seen in neurological disorders, with basic science research demonstrating neuroprotective roles. Treating neurological disorders with adipokine therapy in the clinical setting remains unexplored. This is mainly due to the lack of consensus on adipokine changes in neurological disorders. In the context of AD, there remains a lack of therapy that can efficiently mitigate AD pathology. We propose the use of combination therapy by complementing conventional treatments such as cholinesterase inhibitors with metabolic drugs that manipulate adipokine levels to enhance AD treatment. Chronic donepezil treatment in MCI and AD patients leads to a decrease in BMI and abdominal circumference with lower serum leptin levels and higher serum adiponectin levels [107]. Low-dose galantamine improves oxidative stress, inflammation, insulin resistance, and autonomic regulation in patients with metabolic syndrome [108]. These drugs that were originally designed to target neurological disorders can be adapted to target metabolic disorders. Trending metabolic drugs utilized for obesity and type 2 diabetes mellitus, such as glucagon-like-peptide-1 receptor agonist and metformin, are being repurposed as novel treatment strategies for neurodegenerative diseases [109,110]. These treatments have a direct effect on the brain, as well as an indirect effect by improving overall metabolic health including adipokine profiles. The remodeling of adipokine profiles extend to lifestyle interventions such as diet and exercise, emphasizing both direct and indirect effects on neurological health [111,112]. Dietary choline in early life impacts cognitive function in a familial AD mouse model [113]. Treatment with an adiponectin mimetic rescues memory deficits by ameliorating neuronal insulin resistance in AD mice [114]. It is unlikely that the manipulation of a single adipokine will have a pronounced effect on the brain; rather, the focus should be on achieving a healthy adipose tissue environment, leading to a proper balance of overall adipokine levels. Targeting the adipose tissue to repair vascular damage in AD and other neurological disorders is a novel approach that changes the landscape of viewing and treating CNS disorders [115]. Furthermore, the effects of metabolic treatment on AD should expand from canonical AD pathologies such as amyloid plaques and neurofibrillary tangles to the neuro–glial–vascular landscape encompassing the neurovascular unit, blood-brain barrier, myelin sheath, and glymphatic drainage [116]. Viewing AD and other neurological disorders from a brain–body perspective and treating them by targeting both central and peripheral systems will maximize the efficacy of interventions. It is generally accepted that earlier diagnosis and treatment of AD results in greater effectiveness; therefore, interventions should focus on the midlife weight gain phase and early late life preclinical phase.

## 7. Concluding Remarks

Understanding the brain–body connection in health and disease provides multiple viewpoints and a more comprehensive overview of a pathology compared to historical approaches to diagnosis and treatment. The utilization of adipokines as diagnostic tools and molecular targets for pharmacological treatments in neurological disorders highlights the need to view neurodegeneration with a metabolic lens. Exploring the region-specific effects of adipokines on AD pathology with a temporal component represents the next phase in this field. Instead of manipulating a single adipokine, further research should focus on identifying a comprehensive adipokine atlas in various timepoints of AD pathology. For human studies, longitudinal tracking of the adipokine profile will be more useful than cross-sectional studies due to the vast interindividual variability of AD pathology. In the context of rodent studies, it is crucial to utilize different AD models with various severities of AD pathology. For example, using a rodent model that mimics a slower pace and progression of AD allows better tracking of adipokine profiles in association with AD pathology. To effectively establish the role of adipokines in neurodegeneration, it is essential to identify the mechanisms through which these chemical messengers exert their effects. Adipokines act on various targets, including the neurovascular unit, blood-brain barrier, myelin sheath, and glymphatic system. Adipokine-induced dysfunction in one of these functional neural modules can lead to a domino effect of neural rewiring and impairments. This reinforces the concept that most chronic complications span multiple organs and, to effectively prevent and treat these complex disorders, crosstalk and collaboration between fields are required. Targeting the adipose tissue may be more practical than targeting the brain to impact cognitive function due to the accessibility of peripheral organs. The concept of targeting peripheral organs to treat CNS disorders emphasizes the importance of advancing our knowledge in the brain-body crosstalk.

## Data Availability

Not applicable.
